# Linear Focal Elastosis: What We Know From Epidemiological Studies

**DOI:** 10.1111/ajd.14569

**Published:** 2025-07-18

**Authors:** Tim Aung, Rowland Noakes, Kais Kasem, Dedee F. Murrell

**Affiliations:** ^1^ Bond University Gold Coast Queensland Australia; ^2^ University of Queensland Brisbane Queensland Australia; ^3^ Star Medical Centre‐Woodridge Logan Central Queensland Australia; ^4^ The Skin Lab Brisbane Queensland Australia; ^5^ Queensland Institute of Dermatology Brisbane Queensland Australia; ^6^ Clinical Pathology Department University of Melbourne Melbourne Victoria Australia; ^7^ Faculty of Medicine UNSW Medical School Sydney New South Wales Australia; ^8^ Department of Dermatology St George Hospital Campus Sydney New South Wales Australia

**Keywords:** epidemiology, linear focal elastosis, striae

## Abstract

Linear focal elastosis (LFE), characterised by horizontal streaks on the lower back, is a dermatological condition with unclear etiopathogenesis and limited epidemiological data. This study synthesises case reports to elucidate demographic patterns, clinical manifestations, and potential associations. A literature search across PubMed, Embase, Ovid and ResearchGate identified 37 relevant articles, yielding 80 cases after excluding duplicates and irrelevant articles. In analysis, the male‐to‐female ratio for LFE was 5:1, with a mean age of 39 years. Cases were more common among adolescents and older adults (age ≥ 60), with the majority occurring in pubertal adolescents. No racial predilection was observed. The lower back was the most frequently affected site, with rare cases involving other body parts. Some cases were associated with growth spurts or strenuous exercise, although the etiopathogenesis remains speculative. LFE may be underreported due to its asymptomatic nature and resemblance to other skin conditions, particularly striae distensae. Further research is required to clarify its pathogenesis and explore potential treatment options.

**Summary**

- Why was the study undertaken?
  ○ Since its first report in 1989, the epidemiology of linear focal elastosis (LFE) has not been established on a global scale.
- What does this study add?
  ○ This study offers a scoping review of epidemiological insights revealing a male‐to‐female sex ratio of 5:1 and a high prevalence among pubertal adolescents, 25% of whom link with growth spurts.
- What are the implications of this study for disease understanding and/or clinical care?
  ○ The findings of valuable epidemiological insights will aid future research efforts and improve understanding of the condition.

## 1 Introduction

Linear focal elastosis (LFE), characterised by asymptomatic, horizontal, palpable striae on the lower back, was first reported in 1989 in three older white Caucasian males [1]. Subsequent cases have been identified across various ages, sexes and ethnicities [1, 2]. However, the accurate demographic profile has not been well established in global status. The cause remains unclear, with hypotheses suggesting degenerative–regenerative processes in elastic fibres or excessive fibre regeneration from striae distensae [2]. This literature review aims to provide a comprehensive analysis of LFE epidemiology with new insights based on globally available data.

## 2 Methods

### 2.1 Data Collection

A literature review was conducted with a search term ‘linear focal elastosis’, starting with PubMed and extending to Embase, Ovid and ResearchGate, published up to 30 September 2024. The inclusion criteria were confined to case reports and series of LFE. Exclusion criteria encompassed non‐case‐report/series articles, including those focused on striae distensae, pseudoxanthoma elasticum, mid‐dermal elastolysis or reviews of LFE pathophysiology and cutaneous elastic tissue anomalies. The PubMed search yielded a total of 37 articles, of which 29 met the inclusion criteria following the exclusion of irrelevant studies. Searches in Embase and Ovid yielded no relevant results. To identify further cases published in non‐indexed journals, the search was extended to ResearchGate, which initially returned over 100 articles but lacked functionality for proper extraction. Consequently, we manually reviewed the titles and abstracts of the first 100 articles and identified eight additional eligible studies using the same criteria. The eligible articles, after reviewing full texts, were exported to Excel for analysis. To minimise further duplication bias, case reports from the same authors or regions were excluded, if their case series had already been incorporated. In this process, two case‐report articles were removed, as their cases appeared in two existing case series. Ultimately, a total of 37 articles, including 2 case series, met the inclusion criteria, representing 80 cases. The data were then compiled in a chart (Table S1), with articles numbered 1–29 sourced from PubMed and numbers 30–37 from ResearchGate. Table 1 summarised data from Table S1, to demonstrate the demographic distribution for each category [3, 4, 5, 6, 7, 8, 9, 10, 11, 12, 13, 14, 15, 16, 17, 18, 19, 20, 21, 22, 23, 24, 25, 26, 27, 28, 29, 30, 31, 32, 33, 34, 35, 36, 37, 38, 39].

**TABLE 1:** Summarised data of demographic distribution for linear focal elastosis [3, 4, 5, 6, 7, 8, 9, 10, 11, 12, 13, 14, 15, 16, 17, 18, 19, 20, 21, 22, 23, 24, 25, 26, 27, 28, 29, 30, 31, 32, 33, 34, 35, 36, 37, 38, 39].

Characteristic	Number (*n* = 80)	Proportion (%)
**Sex**		
Male	66	82.5
Female	14	17.5
**Age (years)**		
0–9	1	1.3
10–19	54	67.5
20–29	6	7.5
30–39	3	3.8
40–49	0	0.0
50–59	2	2.5
60–69	2	2.5
70–79	6	7.5
80+	6	7.5
**Ethnicity**		
African/Black	4	5
Middle East	2	2.5
Caucasian	15	18.8
Northeast Asian	38	47.5
South Asian	20	25
Southeast Asian	1	1.3
**Anatomical site**		
Lower back	72	90
Face	2	2.5
Lower limb (Thigh, Knee and Leg)	6	7.5
Arms, Torso and Flank^a^	2	2.5
**Onset duration (*n =* 34)** ^b^		
Less than 1 year	15	44.1
1–2 years	10	29.4
3–5 years	1	2.9
Greater than 5 years	8	23.5

^a^ A study with 2 cases reported involvement of arms, torso and flank along with primary back [12].

^b^ Duration from onset before presentation to clinicians was mentioned in 34 cases only.

### 2.2 Statistical Analysis

Data analysis included calculating mean and median ages, age distribution and male‐to‐female sex ratios in Microsoft Excel. Chi‐square test was applied to assess significance in age distribution and affected body regions.

## 3 Results

### 3.1 Demographics: Age, Sex and Race

Among the 80 cases, 66 (82.5%) were male and 14 (17.5%) were female, resulting in a male‐to‐female ratio of approximately 5:1. Ages ranged from 7 to 93 years, with a mean age of 39 years. Age distribution showed a higher incidence among adolescents (10–19 years, 67.5%, *n* = 54, *p* < 0.01) and older adults (≥ 60 years,17.5%, *n* = 14). The duration from onset to presentation at healthcare providers varied widely from a few months to several decades, with most cases presenting within 1–2 years of onset. This study captured more cases among Asian populations (70%) primarily due to a higher number of publications including case series from Northeast and South Asia. Therefore, this finding may not necessarily imply a higher prevalence in a particular race. Cases encompassed diverse ethnic backgrounds, including Asian, African, Middle Eastern and Caucasian (White) populations.

### 3.2 Clinical Presentation: Site and Morphology

**FIGURE 1:**
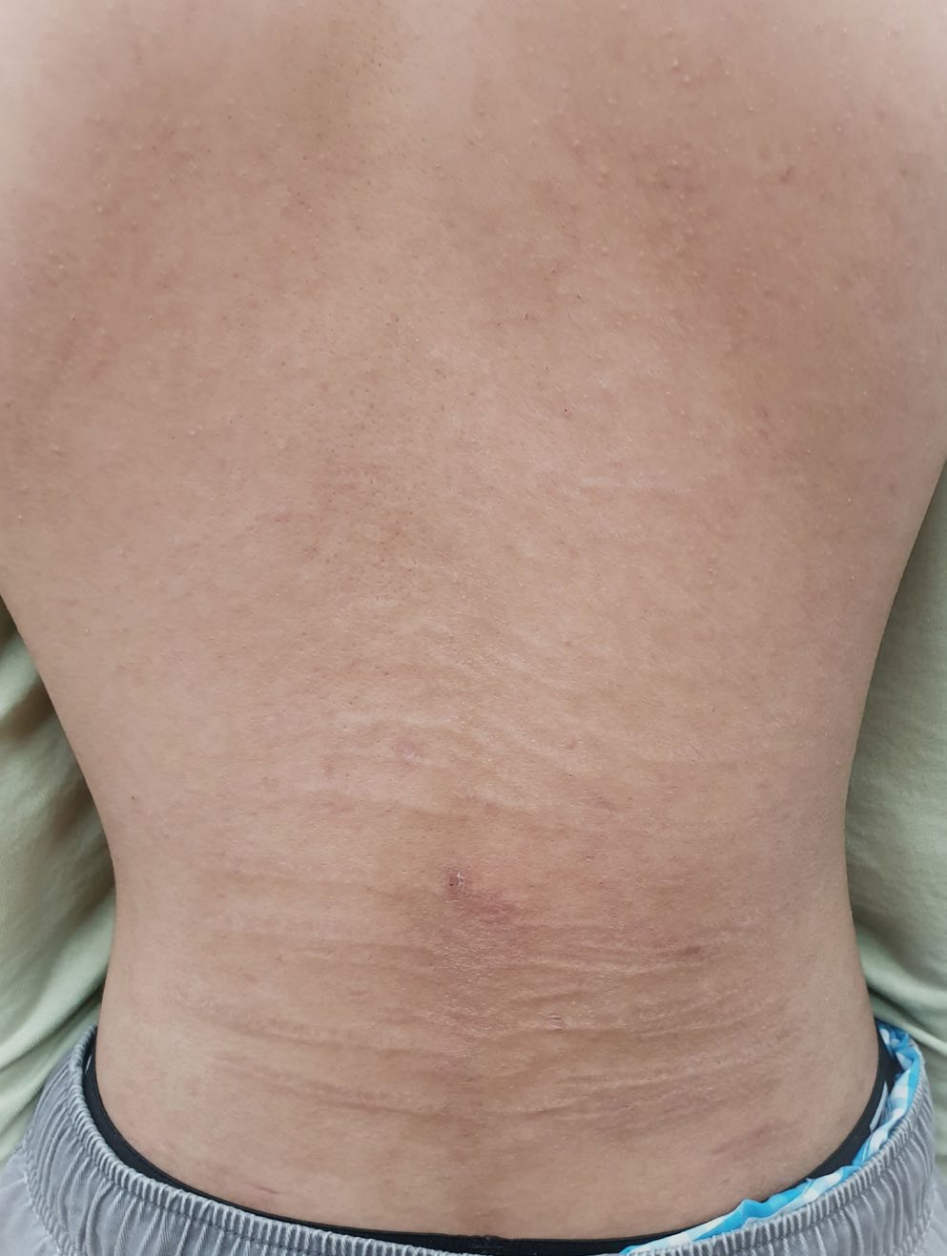
Linear focal elastosis on the lower back of adolescent.

LFE primarily affected the lower back symmetrically in 90% of cases (72 out of 80), with some extending to adjacent mid‐back. Isolated presentations included involvement of the face (2 cases) and lower limbs (thigh, knee or leg‐6 cases). Interestingly, a study with two Black African cases reported presentation after two decades, involving the arms, torso and flank along with the primary back [12]. A Chi‐square test indicated a significant tendency for the lower back (*p* < 0.01). While traditionally described as yellow, palpable bands, some cases presented with white, pink or red streaks, occasionally accompanied by atrophy [6, 12, 32]. This variation may indicate possible early stages or evolving presentation of LFE, warranting further investigation. Figure 1 illustrates a typical case of LFE in an adolescent, involving the lower back with extension to the adjacent mid‐back.

### 3.3 Etiopathology, Associations and Treatment

None of the 37 articles provided a clear understanding of the etiopathogenesis of LFE. The majority consistently described the cause as ‘unknown’ or ‘unclear’, with a commonly cited theory proposing that the condition involves a degenerative‐regenerative process of elastic fibres or excessive regenerative changes originating from striae distensae (SD). One study suggested that LFE may be preceded by SD in its pathogenesis [10]. However, this review found that LFE was linked to growth spurts in adolescence (aged 10–19) in 25% of cases [6, 9, 32] and intense physical activity, such as swimming and running, in 5% of cases [5, 8, 32]. Family history was recorded in 3 out of 80 (3.7%) [6, 28, 34], which seems insufficient to suggest a genetic predisposition. Association or coexistence with striae distensae (SD) was described in five studies [9, 19, 20, 23, 29], and misdiagnosis with SD was reported in three cases [7, 34]. Several studies described underestimation or underreporting of LFE due to its asymptomatic nature and clinical similarity with SD [6, 7, 26, 32, 34]. Treatment trials involving topical & intralesional corticosteroids, topical retinoids, centella asiatica extract and platelet‐rich plasma (PRP) were mentioned in two case series and other case reports, but yielded no satisfactory improvement [1, 6, 32]. The majority of reports characterised LFE as asymptomatic and benign, with no explicit mention of long‐term outcome (prognosis).

## 4 Discussion

The findings suggest that LFE is more common in males, with incidence peaking during adolescence and older adulthood (age ≥ 60). Overall, the majority of cases occur in adolescents, some of which are associated with growth spurts. The condition's asymptomatic nature, combined with its resemblance to striae distensae (SD), likely contributes to underreporting and diagnostic delays. However, LFE should be distinguishable from SD based on the latter's clinical characteristics—morphology, typical sites of involvement (e.g., abdomen, gluteal region and proximal extremities), and associated risk factors (e.g., obesity, pregnancy or steroid use), as well as histological findings—elastolysis in the acute stage, with later stages exhibiting epidermal atrophy and densely packed, thin, horizontally arranged collagen bundles within the dermis [1, 40].

Although theories on elastic fibre degeneration exist, the exact pathophysiology remains unclear. Factors like growth spurts and strenuous physical activities may play a role in triggering or exacerbating LFE, particularly during adolescence. Further research into the molecular basis of LFE may provide insights into its cause and potential therapeutic approaches.

Limitations: This study may be limited by its small sample size (*n* = 80) based on case reports and series rather than population‐based studies, and restriction to English‐language articles only. Additionally, certain platforms such as ResearchGate lack the necessary structure for proper data extraction and Google Scholar was not considered. However, the global availability of cases through reputable databases provides valuable epidemiological insights. To the best of our knowledge, only two single‐institution studies have been conducted [6, 32], with no comprehensive national or global‐level analyses. As such, this review may offer a broader perspective on the epidemiology of linear focal elastosis.

## 5 Conclusion

Linear focal elastosis is a sporadic and possibly underrecognised dermatological condition, with a higher prevalence among pubertal adolescents, and a male‐to‐female ratio of 5:1. It has no clearly observed racial predilection, and typically appears on the lower back, with potential extension to mid‐back. Its benign, asymptomatic nature and resemblance to striae distensae may contribute to underreporting. Further research is needed to understand its etiopathogenesis and to explore effective treatment options.

## Ethics Statement

The authors have nothing to report.

## Conflicts of Interest

The authors declare no conflicts of interest.

## Supporting information


**Table S1.** Linear focal elastosis study plot chart.

## Data Availability

The data that support the findings of this study are available on request.
