# Supplementary material for: Linear Focal Elastosis: What We Know From Epidemiological Studies
Source: Australas J Dermatol. 2025 Jul 18;66(6):364–8. doi: 10.1111/ajd.14569 (PMC12418147; doi:10.1111/ajd.14569)
Supplement: Supplementary file 1 — Table S1. Linear focal elastosis study plot chart. [file AJD-66-364-s001.zip › ajd14569-sup-0001-TableS1.docx]

**Table 1. Linear focal elastosis study plot chart (supplementary)**

|  | **Author and publication year** | **No. of cases** | **M** | **F** | **Age at presen-tation** | **Duration from onset (year)** | **Race** | **Site** | **Association/ Comments** |
| --- | --- | --- | --- | --- | --- | --- | --- | --- | --- |
| 1 | Kim et al (2022)^3^ | 1 | 0 | 1 | 93 | 2 | NE Asian | Face | oldest case with whitish, atrophic |
| 2 | Garg et al (2021)^4^ | 1 | 1 | 0 | 12 | x | S Asian | L Back | x |
| 3 | Kaur et al (2019)^5^ | 1 | 0 | 1 | 16 | 3 | S Asian | Knees | marathon runner |
| 4 | Seol et al (2019)^6^ | 22 | 19 | 3 | 13.5^*^  (12 – 19) | x | NE Asian | L Back | 45.5% (growth spurt), a case of FHx, underestimate |
| 5 | Zink et al (2018)^7^ | 1 | 0 | 1 | 58 | 30 | White | L Back | underreport and delay due to misdiagnosis with SD |
| 6 | Florell et al (2017)^8^ | 1 | 1 | 0 | 60 | 1 | White | L Back | exercise (swimming) |
| 7 | Jang et al (2012)^9^ | 1 | 1 | 0 | 15 | x | NE Asian | L Back | growth spurt, coexistence with SD |
| 8 | Jeong et al (2011)^10^ | 1 | 1 | 0 | 14 | 2 | NE Asian | L Back | LFE follow SD |
| 9 | Adişen et al (2007)^11^ | 1 | 1 | 0 | 14 | 0.25 | Mid-East | L Back | x |
| 10 | Whalen et al (2006)^12^ | 2 | 1 | 1 | 35.5^*^ | 20 | African | L Back | Also on arm, torso & flanks; can be yellow, red or white |
| 11 | Pui et al (2003)^13^ | 1 | 1 | 0 | 83 | 1 | White | L Back | red-yellow |
| 12 | Inaloz et al (2003)^14^ | 1 | 1 | 0 | 50 | 2 | Mid-East | face | x |
| 13 | Arroyo et al (2001)^15^ | 1 | 1 | 0 | 83 | 2 | White | L Back | x |
| 14 | Akagi et al (2002)^16^ | 1 | 0 | 1 | 89 | x | NE Asian | T & L | coexistence with PXE |
| 15 | Ramlogan et al (2001)^17^ | 1 | 0 | 1 | 13 | 2 | White | T & L | x |
| 16 | Choi et al (2000)^18^ | 2 | 2 | 0 | 17^*^ | 2 | NE Asian | L Back | shoulder involvement in one case |
| 17 | Parsad (1998)^19^ | 1 | 1 | 0 | 19 | 2 | S Asian | L Back | SD over axilla |
| 18 | Hashimoto (1998)^20^ | 1 | 1 | 0 | 32 | x | African | L Back | SD on shoulders and buttocks |
| 19 | Chang et al (1998)^21^ | 2 | 2 | 0 | 10.5 | x | NE Asian | L Back | aged 7 & 14 |
| 20 | Breier et al (1997)^22^ | 1 | 0 | 1 | 13 | 1.5 | SE Asian | Legs | x |
| 21 | Hagari et al (1997)^23^ | 1 | 0 | 1 | 73 | x | NE Asian | L Back | associated SD |
| 22 | Tamada et al (1997)^24^ | 3 | 3 | 0 | 22* | 12 | NE Asian | L Back | x |
| 23 | Palmer et al (1995)^25^ | 1 | 1 | 0 | 61 | 42 | White | L Back | since aged 19 |
| 24 | Vogel et al (1995)^26^ | 2 | 2 | 0 | 79^*^ | x | White | L Back | aged 69 & 89, underreport |
| 25 | Trüeb et al (1995)^27^ | 1 | 0 | 1 | x | x | White | L Back | x |
| 26 | Moiin et al (1994)^28^ | 1 | 1 | 0 | 29 | x | African | L Back | since childhood, FHx in father |
| 27 | White (1992)^29^ | 1 | x | x | x | x | White | L Back | SD both axillae |
| 28 | Hagari et al (1991)^30^ | 1 | 1 | 0 | 86 | 26 | NE Asian | L Back | x |
| 29 | Burket et al (1989)^31^ | 3 | 3 | 0 | 71.5* | x | White | L Back | x |
| 30 | Palaniappan et al  (2024)^32^ | 14 | 14 | 0 | 15.22*  (11 – 19) | 0.75 | S Asian | L Back | growth spurt (21.4%), exercise (14.3%), atrophic in 10, underestimate |
| 31 | Clement et al (2018)^33^ | 1 | 1 | 0 | 20 | x | White | L Back | joint hypermobility |
| 32 | Gupta (2017)^34^ | 2 | 2 | 0 | 17 | x | S Asian | L Back | misdiagnosed with SD, FHx, underreport |
| 33 | Huang et al (2008)^35^ | 1 | 1 | 0 | 17 | x | S Asian | L Back | x |
| 34 | Brennard et al (2007)^36^ | 1 | 1 | 0 | 84 | x | White | L Back | x |
| 35 | Shivas et al (2007)^37^ | 1 | 1 | 1 | 16 | x | S Asian | L Back | x |
| 36 | Kim et al (2006)^38^ | 1 | 0 | 1 | 13 | x | NE Asian | Thighs | x |
| 37 | Lee et al (2006)^39^ | 1 | 1 | 0 | 22 | x | NE Asian | Legs | coexist with psoriasis |
|  | **Total** | **80** | **66** | **14** | **1366** |  |  | **72 (lower back)** | |
|  |  |  | **4.7:** | **1** | **39.03 (mean)** | |  |  |  |
| Abbreviations: No, number; M, male; F, female; NE Asian, Northeast Asian; SE Asian, Southeast Asian; S Asian, South Asian; Mid-East, Middle Eastern; L Back, lower back; T & L, thighs and legs; FHx, family history; SD, striae distensae; PXE, Pseudoxanthoma elasticum; x, unavailable data; *, Mean age was calculated and utilized for those reports with greater than a single case or case series. | | | | | | | | | |
