# Supplementary material for: Linear Focal Elastosis: What We Know From Epidemiological Studies
Source: Australas J Dermatol. 2025 Jul 18;66(6):364–8. doi: 10.1111/ajd.14569 (PMC12418147; doi:10.1111/ajd.14569)
Supplement: Supplementary file 1 — Table S1. Linear focal elastosis study plot chart. [file AJD-66-364-s001.zip › Supplementary_continued references.docx]

**Supplementary (continued references)** “Linear focal elastosis: What we know from epidemiological studies” [AJD Manuscript ID: 5630599]

1. Adişen E, Ilter N, Erdem O, Gürer MA. Early onset linear focal elastosis in a Turkish boy. *Turk J Pediatr*. 2007 Oct-Dec;49(4):441-3.
2. Whalen JG, English JC 3rd. Case study on linear focal elastosis. *Dermatol Nurs*. 2006 Oct;18(5):469-71.
3. Pui JC, Arroyo M, Heintz P. Linear focal elastosis: histopathologic diagnosis of an uncommon dermal elastosis. *J Drugs Dermatol*. 2003 Jan;2(1):79-83.
4. Inaloz HS, Kirtak N, Karakok M, Ozgoztasi O. Facial linear focal elastosis: a case report. *Int J Dermatol*. 2003 Jul;42(7):558-60. doi: 10.1046/j.1365-4362.2003.01733.x.
5. Arroyo MP, Soter NA. Linear focal elastosis. *Dermatol Online J*. 2001 Dec;7(2):18.
6. Akagi A, Tajima S, Kawada A, Ishibashi A. Coexistence of pseudoxanthoma elasticum-like papillary dermal elastolysis and linear focal dermal elastosis. *J Am Acad Dermatol*. 2002 Aug;47(2 Suppl):S189-92. doi: 10.1067/mjd.2002.108495.
7. Ramlogan D, Tan BB, Garrido M. Linear focal elastosis. *Br J Dermatol*. 2001 Jul;145(1):188-90. doi: 10.1046/j.1365-2133.2001.04319.x.
8. Choi SW, Lee JH, Woo HJ, Park CJ, Yi JY, Song KY, Kim HO. Two cases of linear focal elastosis: different histopathologic findings. *Int J Dermatol*. 2000 Mar;39(3):207-9. doi: 10.1046/j.1365-4362.2000.00848.x.
9. Parsad D. Linear focal elastosis. *Indian J Dermatol Venereol Leprol*. 1998 Nov-Dec;64(6):299-300.
10. Hashimoto K. Linear focal elastosis: Keloidal repair of striae distensae. *J Am Acad Dermatol*. 1998 Aug;39(2 Pt 2):309-13. doi: 10.1016/s0190-9622(98)70378-0.
11. Chang SE, Park IJ, Moon KC, Koh JK. Two cases of linear focal elastosis (elastotic striae). *J Dermatol*. 1998 Jun;25(6):395-9.
12. Breier F, Trautinger F, Jurecka W, Hönigsmann H. Linear focal elastosis (elastotic striae): increased number of elastic fibres determined by a video measuring system. *Br J Dermatol*. 1997 Dec;137(6):955-7.
13. Hagari Y, Norimoto M, Mihara M. Linear focal elastosis associated with striae distensae in an elderly woman. *Cutis*. 1997 Nov;60(5):246-8; quiz 250.
14. Tamada Y, Yokochi K, Ikeya T, Nakagomi Y, Miyake T, Hara K. Linear focal elastosis: a review of three cases in young Japanese men. *J Am Acad Dermatol*. 1997 Feb;36(2 Pt 2):301-3. doi: 10.1016/s0190-9622(97)80403-3.
15. Palmer J, Madison KC, Stone MS. Asymptomatic linear bands across the back. Linear focal elastosis. *Arch Dermatol*. 1995 Sep;131(9):1070-1, 1073-4. doi: 10.1001/archderm.131.9.1070.
16. Vogel PS, Cardenas A, Ross EV, Cobb MW, Sau P, James WD. Linear focal elastosis. *Arch Dermatol*. 1995 Jul;131(7):855-6. doi: 10.1001/archderm.131.7.855.
17. Trüeb RM, Fellas AS. Lineäre fokale Elastose (Elastotische Striae) [Linear focal elastosis (elastotic striae)]. *Hautarzt*. 1995 May;46(5):346-8. German. doi: 10.1007/s001050050265.
18. Moiin A, Hashimoto K. Linear focal elastosis in a young black man: a new presentation. *J Am Acad Dermatol*. 1994 May;30(5 Pt 2):874-7. doi: 10.1016/s0190-9622(94)70103-2.
19. White GM. Linear focal elastosis: a degenerative or regenerative process of striae distenae? *J Am Acad Dermatol*. 1992 Sep;27(3):468. doi: 10.1016/s0190-9622(08)80889-4.
20. Hagari Y, Mihara M, Morimura T, Shimao S. Linear focal elastosis. An ultrastructural study. *Arch Dermatol*. 1991 Sep;127(9):1365-8.
21. Burket JM, Zelickson AS, Padilla RS. Linear focal elastosis (elastotic striae). *J Am Acad Dermatol*. 1989 Apr;20(4):633-6. doi: 10.1016/s0190-9622(89)70075-x.
22. Palaniappan V, Selvaarasan J, Kaliaperumal K. A retrospective study of linear focal elastosis among adolescents. *Indian J Paediatr Dermatol*. 2024;25:267-9.
23. Clement BC, Forster C, Logemann N. Focal linear elastosis in a patient with joint hypermobility syndrome. *Dermatol Online J*. 2018 Jan 15;24(1):13030/qt6n9456xv.
24. Gupta, M. Linear Focal Elastosis in a Family. *JMSCR*. 2017 Aug; 05 (08): 27220-23. doi:10.18535/JMSCR/V5I8.205
25. Huang T M, Lee JY, Yang CC. (2008). Linear Focal Elastosis-A Case Report. *Dermatologica Sinica*. 2008 Jun; 26(2), 99-106. doi.org/10.29784/DS.200806.0007
26. Brennand S, Kalnins R. Linear focal elastosis. *Australas J Dermatol*. 2007 Oct; 48 (4): 144. doi: 10.1111/j.1440-0960.2007.00408_5.x.
27. Shivaswamy KN, Babu A, ThappaIndian DM. Linear focal elastosis (Elastosis striae). *Indian J Dermatol*. 2007:52(1):66-67.
28. Kim YY, Kim MY, Park YM, Kim HO. A Case of Linear Focal Elastosis on Both Lateral Thighs. *Korean J Dermatol*. 2006;44(3):326-329.
29. Lee WS, Choi MJ, Kang H. A Case of Linear Focal Elastosis on the Lower Extremities. *Korean J Dermatol*. 2006;44(8):1010-1012.
30. MacGregor JL, Wesley NO. Striae distensae (stretch marks). In Jeffrey Dover & Abena Ofori (Ed.), *UpToDate*, 2024. Available from https://www.uptodate.com/contents/striae-distensae-stretch-marks; Accessed 18 Apr 2025.

**----------------**
